# Non-professional caregiver burden is associated with the severity of patients’ cognitive impairment

**DOI:** 10.1371/journal.pone.0204110

**Published:** 2018-12-06

**Authors:** Christopher M. Black, Craig W. Ritchie, Rezaul K. Khandker, Robert Wood, Eddie Jones, Xiaohan Hu, Baishali M. Ambegaonkar

**Affiliations:** 1 Center for Observational and Real-World Evidence, Merck & Co., Inc., Kenilworth, New Jersey, United States of America; 2 Centre for Dementia Prevention, Centre for Clinical Brain Sciences, University of Edinburgh, Edinburgh, United Kingdom; 3 Adelphi Real World, Macclesfield, United Kingdom; 4 University of Southern California, Los Angeles, California, United States of America; Nathan S Kline Institute, UNITED STATES

## Abstract

**Background/Objectives:**

To analyse the relationship between caregiver burden and severity of patients’ cognitive impairment.

**Design:**

Data were drawn from the cross-sectional 2015/2016 Adelphi Real World Dementia Disease-Specific Programme.

**Setting:**

This research was multi-national and studied physicians and their consulting patients with cognitive impairment.

**Participants:**

1,201 caregivers completed self-assessment forms.

**Measurements:**

Validated instruments of caregiver wellbeing and burden (EQ-5D-3L questionnaire, EQ-VAS, Zarit Burden Interview, and Work Productivity and Activity Impairment questionnaire) and number of caregiver hours were analysed by severity of patients’ cognitive impairment, categorised according to the Mini-Mental State Examination. Data were analysed using Spearman’s rank correlation coefficients and ordinary least squares regression models, to compare outcomes between caregivers of patients with prodromal, mild, moderate, and severe dementia.

**Results:**

The majority of caregivers were female (69.1%), lived with the patient they cared for (75.8%), and only approximately one third (28.3%) were in part- or full-time employment. There were statistically significant (p<0.001) increases in caregiver time (36.9 versus 108.6 hours per week for prodromal versus severe dementia, respectively) and measures of caregiver burden and health status (EQ-5D-3L, EQ-VAS, and Zarit Burden Interview) and increases in measures of work productivity and activity impairment with increasing severity of patients’ disease.

**Conclusion:**

This study of real-world data confirmed an association between increased caregiver burden and severity of patients’ cognitive impairment by analysis of a wide range of validated measures of caregiver burden. These findings suggest that maintaining patients in the earliest stages of their disease for as long as possible may potentially help to protect caregiver wellbeing, although further research is required to confirm this hypothesis.

## Introduction

Worldwide, more than 35 million people live with dementia and this is predicted to increase to 115 million by 2050 [1]. Patients with neurodegenerative diseases typically experience progressive cognitive impairment (CI) and develop severe manifestations including dementia, of which the most frequent aetiology is Alzheimer’s disease (AD), representing up to 75% of all cases of dementia [2–5].

Dementia has a considerable negative impact on patients’ family members, placing physical, emotional, and financial strains on these non-professional caregivers [6]. Indeed, the family caregiver has been referred to as ‘a second patient in the making’ and evidence suggests that the burden on caregivers of dementia patients is particularly high [7]. In an analysis of data from more than 1500 family caregivers, dementia caregivers (n = 320) spent significantly more hours per week providing care and reported greater impacts in terms of employment complications, strain, mental and physical health problems, time for leisure and family, and family conflict, compared with non-dementia carers [8]. Worldwide dementia costs in 2010 were estimated at ~US$600 billion and in high-income countries 45% of total costs were informal care costs [9]. In the GERAS study, an observational study of costs and resource use in 1497 outpatients with mild to severe AD outpatients in France, Germany, and the UK, informal caregiver costs accounted for up to 60% of total societal costs [10].

There is some limited evidence that pharmacological management of patients’ symptoms may be associated with lower levels of caregiver burden [11,12,13]. However, in the absence of treatments to cure or slow disease progression, dementia–and in particular AD dementia–will continue to place a high burden on caregivers and society [14,15].

Various programmes and services have been developed, most notably in high-income countries, to assist family caregivers, including counselling, psychoeducational programmes, and specialised skills training [6]. There is limited evidence that some multicomponent caregiver interventions can help improve health status and decrease burden of caregivers and also enable caregivers to provide at-home care for longer thus delaying nursing home admission for patients [16–18].

Due to the magnitude of the challenge faced, it is important to target interventions where they are most effective, and disease severity appears to be an important factor. Germain et al., 2009 [19] identified severity of CI as an important and significant explanatory variable for caregiver burden on the basis of data from the Impact of Cholinergic Treatment Use (ICTUS) study, a European longitudinal study of 1091 mild to moderate AD patients. The GERAS study also showed that mean time spent caregiving and burden increased significantly with increasing severity of disease [10,20]. Furthermore, in an analysis of 421 US dementia outpatients, severity of patients’ psychiatric symptoms, behavioural disturbances and quality-of-life correlated with caregiver burden; the authors concluded that psychosocial and pharmacologic interventions may not only help to alleviate patient suffering but also help promote caregiver wellbeing [21].

The current study was undertaken to further characterise the relationship between disease severity and caregiver burden in the context of an up-to-date analysis of a large body of real-world data provided by caregivers of patients with all stages of severity of CI using a number of different validated measures of caregiver burden to provide a comprehensive picture.

## Materials and methods

Data were drawn from the 2015/2016 Adelphi Real World Dementia Disease Specific Programme (DSP), a large cross-sectional survey of physicians and their consulting patients with CI, which was conducted in France, Germany, Italy, Spain, the UK, and the US. The full DSP methodology has been published previously [22].

Local fieldwork teams identified target physicians from publicly available lists of healthcare professionals, who were then invited to participate upon fulfilment of predefined inclusion criteria as follows. To be considered, the physicians’ primary speciality was required to be primary care physician, geriatrician, neurologist, psychogeriatrician, psychiatrist, neuropsychiatrist, or neurodegenerative diseases specialist. Participating physicians were required to have received medical qualification between 1979 and 2012 and to be personally responsible for treatment decisions for patients with CI. To ensure sufficient data were collected to meet the DSP requirements, specialist physicians were required to see ≥10 CI patients and primary care physicians were required to see ≥5 CI patients in a typical week.

Physicians were requested to complete a record form for each of their next 10 consecutively consulting CI patients, therefore providing a representative ‘point in time’ sample of consulting patients. For each patient recruited, the physician provided data on the patient’s basic demographics, clinical characteristics including diagnosis, most recent Mini-Mental State Examination (MMSE) score, concomitant conditions and medications, and non-professional caregiver hours required per week. Patients were required to be aged 50 years and over and have a diagnosis of early cognitive impairment or AD. Patients were categorised according to their MMSE score: prodromal (27–30), mild (21–26), moderate (10–20), and severe (<10) dementia [23]. When accompanying the patient to their consultation, caregivers of these same patients were invited to complete a form to capture their demographics, relationship to the patient being cared for, and data from multiple validated instruments of caregiver wellbeing and burden. The EuroQol 5-dimensions 3-level (EQ-5D-3L) questionnaire, which consists of two elements designed for self-completion: the EQ-5D descriptive system and the EQ visual analogue scale (EQ-VAS) [24]. The descriptive system comprises measures of mobility, ability to perform activities of self-care (eg, washing and dressing), ‘usual’ activities (eg, work, study, housework, family and leisure activities), and levels of pain/discomfort and anxiety/depression [25]. Each of these dimensions is divided into three levels of perceived problems: Level 1 indicating no problems, Level 2 indicating some problems, and Level 3 indicating extreme problems; the subject is asked to indicate his/her health state using a tick box method. By combining the five responses; a total of 243 possible health states can be defined. Health states were converted into a utility index using country specific tariffs, where a score of 1 indicates perfect health, a score of 0 indicates death and a score less than 0 indicates a health state worse than death. The EQ-VAS is a single index value for health status which records the caregiver’s self-rated health using a 100-point vertical visual scale ranging from “worst imaginable health state” (0) to “best imaginable health state” (100) [25]. The Zarit Burden Interview (ZBI) assesses caregiver perceptions of burden including the physical, psychological, social, and emotional impact their caring role has on their life and work [26,27]. The full revised version comprises a 22-item subjectively worded self-report inventory, and the caregiver responds to each item using a 5-point Likert scale ranging from ‘never’ (0) to ‘nearly always’ (4). Total scores range from 0 to 88, with higher scores indicating a higher level of perceived burden. The Work Productivity and Activity Impairment (WPAI) questionnaire measures the impact that the caregiver’s role has on levels of absenteeism (work time missed), presenteeism (impairment at work/reduced on-the-job effectiveness), work productivity loss (overall work impairment/absenteeism plus presenteeism), and activity impairment during the last 7 days [28,29]. It consists of six questions concerning employment status, hours of work missed due to caregiving responsibilities, hours of work missed for any other reasons e.g. a vacation, hours actually worked, and the degree to which caregiving responsibilities affected productivity at work and regular daily activities (both measured on a VAS ranging from ‘no effect’ [0] to ‘completely prevented activity’ [10]). The four domains (absenteeism, presenteeism, work productivity loss and activity impairment) are measured from 0 to 100 percentage impairment.

Outcomes were compared between caregivers of patients with prodromal, mild, moderate, and severe dementia. Statistical significance was assessed initially for unadjusted outcome data using Spearman’s rank correlation coefficients due to the ordered nature of the dementia subgroups. Ordinary least square regression models were also constructed for each outcome. Due to the limited number of employed caregivers who could therefore complete the WPAI, only activity impairment was included in the regression analysis. The exposure variable CI subgroup was included as a set of ordinal dummy variables to allow the incremental change in outcome to be assessed between dementia levels. All models included the following covariates: caregivers’ age, sex, and employment status; patients’ body mass index, concomitant cardiovascular and cerebrovascular conditions, and number of concomitant medications; relationship of caregiver to patient; and country. Analyses were conducted in Stata v14.2 or later [30] to a 0.05 significance level.

### Ethics statement

Using a check box, patients and caregivers provided written informed consent for use of their anonymised and aggregated data for research and publication in scientific journals. Data were collected in such a way that patients, caregivers and physicians could not be identified directly; all data were aggregated and de-identified before receipt.

Data collection was undertaken in line with European Pharmaceutical Marketing Research Association guidelines and as such it does not require ethics committee approval. Each survey was performed in full accordance with relevant legislation at the time of data collection, including the US Health Insurance Portability and Accountability Act 1996, and Health Information Technology for Economic and Clinical Health Act legislation.

## Results

In total, 1,201 caregivers volunteered to complete self-assessment forms (Table 1). Approximately half (600/1,201; 50.0%) and one third (436/1,201; 36.3%) of forms were from caregivers of patients with moderate or mild dementia, respectively. Considerably fewer forms were collected from caregivers of prodromal (75/1,201; 6.2%) or severe (90/1,201; 7.5%) dementia patients.

**Table 1:** Caregiver demographics.

	Overall (n = 1,201)	Prodromal (n = 75)	Mild (n = 436)	Moderate (n = 600)	Severe (n = 90)
**Caregiver age**^a^, **years**					
Mean (SD)	63.9 (13.7)	65.0 (12.5)	64.1 (14.0)	63.4 (13.6)	65.8 (13.3)
Median (IQR)	66.0 (54.0–75.0)	66.0 (58.0–74.0)	66.0 (55.0–75.0)	65.0 (53.0–75.0)	67.5 (57.0–76.0)
**Female caregiver**^b^	829 (69.1)	55 (73.3)	299 (68.6)	413 (68.9)	62 (69.7)
**Caregiver lives with patient**^c^	853 (75.8)	54 (77.1)	308 (75.7)	414 (74.1)	77 (86.5)
**Employment status of caregiver**^d^					
Working full time	208 (17.4)	10 (13.3)	77 (17.7)	104 (17.5)	17 (18.9)
Working part time	130 (10.9)	11 (14.7)	49 (11.3)	61 (10.3)	9 (10.0)
Student	11 (0.9)	0 (0)	6 (1.4)	4 (0.7)	1 (1.1)
Homemaker	170 (14.2)	12 (16.0)	54 (12.4)	86 (14.5)	18 (20.0)
Retired	591 (49.5)	38 (50.7)	228 (52.4)	290 (48.9)	35 (38.9)
Unemployed	74 (6.2)	4 (5.3)	18 (4.1)	42 (7.1)	10 (11.1)
Other	9 (0.8)	0 (0)	3 (0.7)	6 (1.0)	0 (0)
**Relationship of caregiver to patient**^e^					
Partner/Spouse	671 (56.8)	52 (70.3)	253 (59.5)	316 (53.3)	50 (55.6)
Female^f^	407 (60.8)	32 (61.5)	152 (60.1)	191 (60.6)	32 (65.3)
Male^f^	262 (39.2)	20 (38.5)	101 (39.9)	124 (39.4)	17 (34.7)
Sibling	30 (2.5)	3 (4.1)	9 (2.1)	14 (2.4)	4 (4.4)
Son	95 (8.0)	1 (1.4)	34 (8.0)	51 (8.6)	9 (10.0)
Daughter	291 (24.6)	12 (16.2)	100 (23.5)	157 (26.5)	22 (24.4)
Other family member	56 (4.7)	4 (5.4)	18 (4.2)	29 (4.9)	5 (5.6)
Friend/neighbour	25 (2.1)	1 (1.4)	7 (1.6)	17 (2.9)	0 (0)
Other relationship	14 (1.2)	1 (1.4)	4 (0.9)	9 (1.5)	0 (0)

Data are n (%) unless otherwise indicated. SD, Standard Deviation; IQR, Interquartile Range

^a^ Overall, 6 missing responses; Mild, 1; Moderate 5.

^b^ Overall, 2 missing responses; Moderate, 1; Severe, 1.

^c^ Overall, 76 missing responses; Prodromal, 5; Mild, 29; Moderate, 41; Severe, 1.

^d^ Overall, 8 missing responses; Mild, 1; Moderate, 7.

^e^ Overall, 19 missing responses; Prodromal, 1; Mild, 11; Moderate, 7.

^f^ As a percentage of all partner/spouse caregivers

Mean caregiver age was 63.9 years and most (69.1%) were female and lived with the patient they cared for (75.8%; Table 1). More than half of caregivers were retired (49.5%) or unemployed (6.2%), with only approximately one third (28.3%) in full- or part-time employment. Most caregivers were a patient’s partner/spouse (56.8%), daughter (24.6%), or son (8.0%). Overall, 60.8% of spousal caregivers were female, ranging from 60.1% in the mild subgroup to 65.3% in the severe subgroup.

### Raw, unadjusted caregiver outcomes

**Fig 1:**
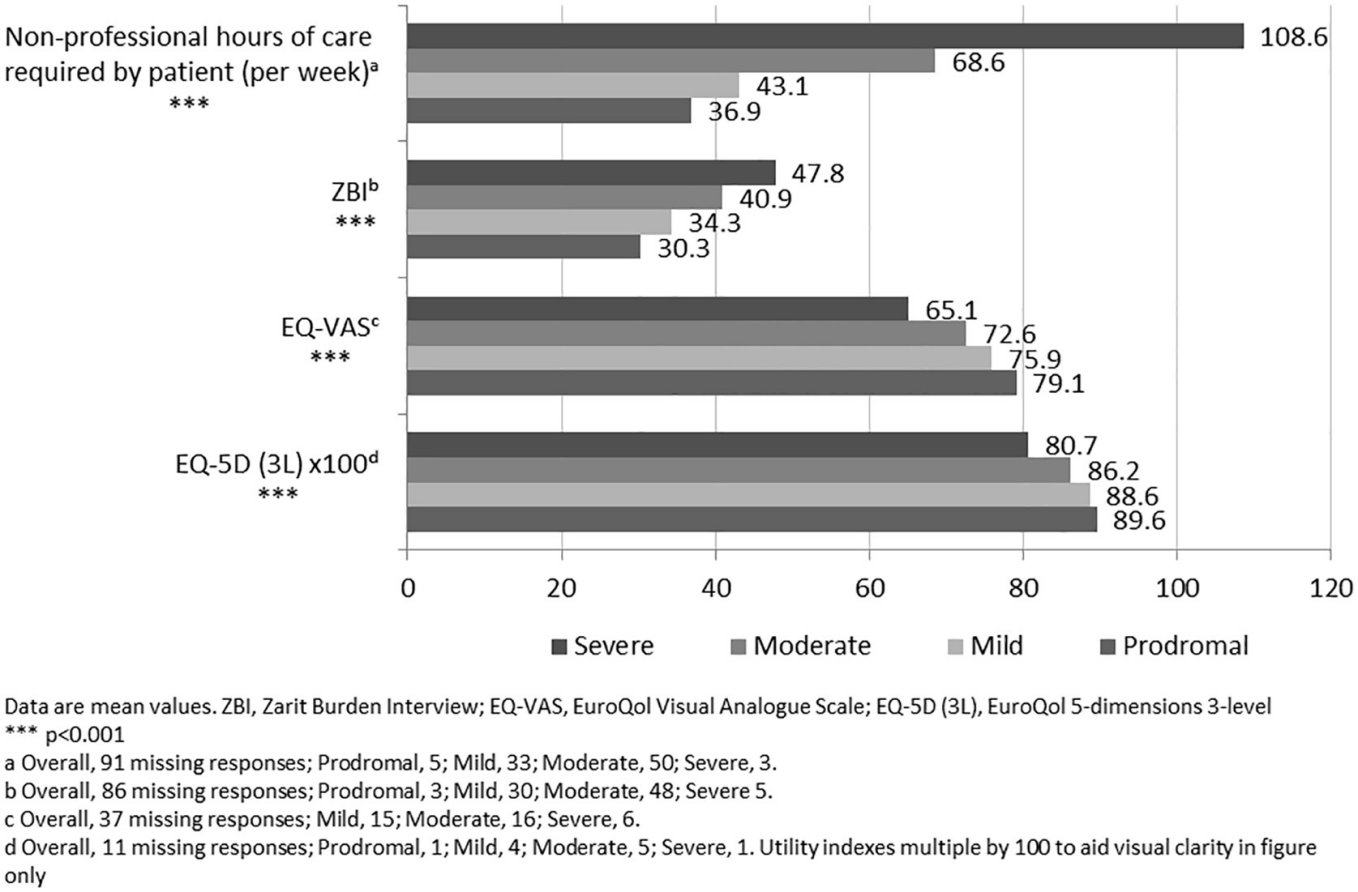
Caregiver outcomes (hours, ZBI, EQ-VAS, EQ-5D-3L) by patient dementia severity.

There was a marked, statistically significant (p<0.001) increase in non-professional caregiver time required per week with increased severity of patients’ disease, from a mean of 36.9 hours for carers of patients with prodromal dementia to 108.6 hours for carers of patients with severe dementia (Fig 1). ZBI scores also increased significantly (p<0.001) with increasing severity of patients’ dementia. In addition, caregiver-reported results of the EQ-5D-3L questionnaire (p<0.001) and EQ-VAS (p<0.001) indicated statistically significant deterioration in caregivers’ health state and perception of health status with increasing patient dementia severity.

**Fig 2:**
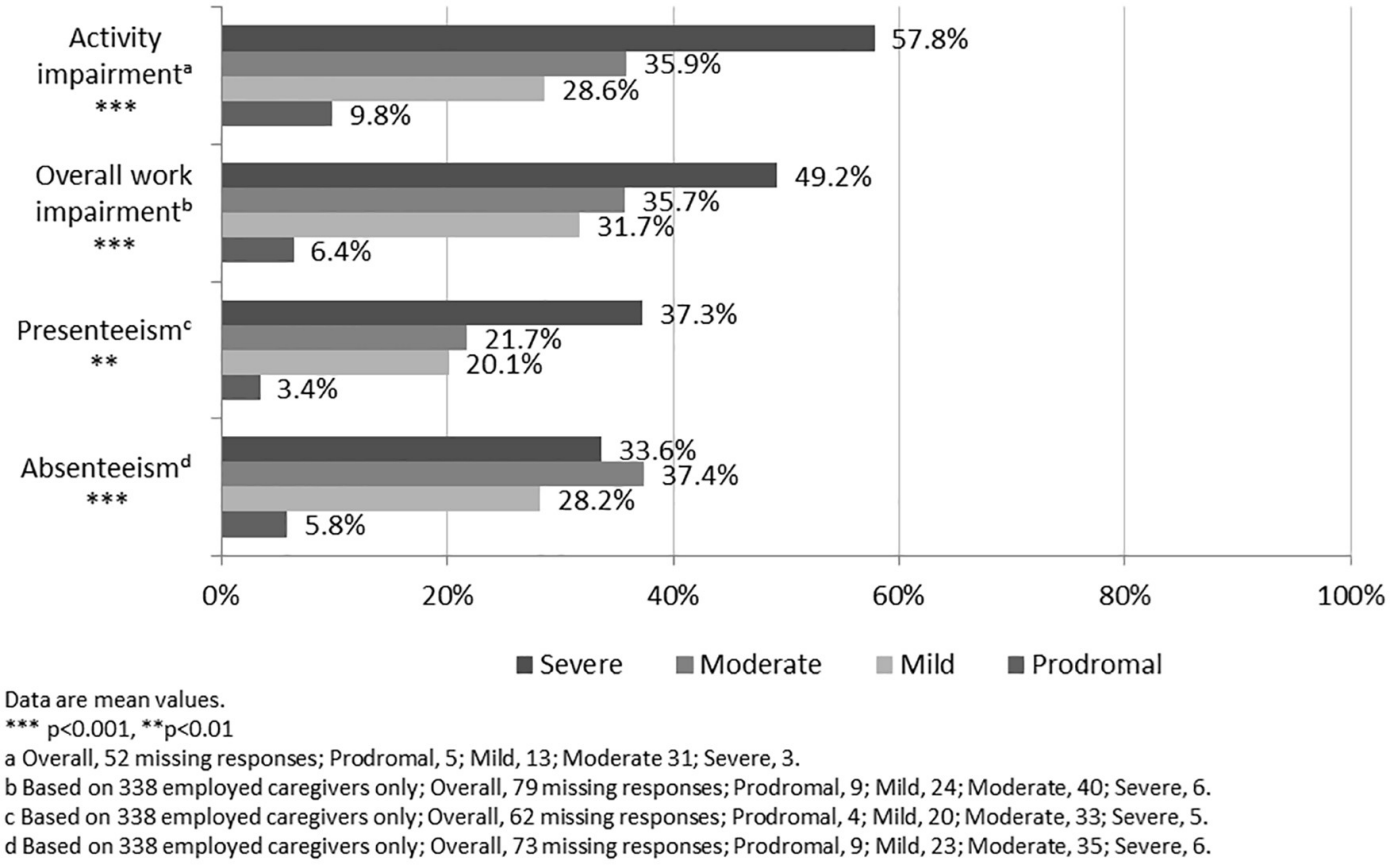
Caregiver outcomes (WPAI questionnaire) by patient dementia severity.

Results from the WPAI questionnaire showed a clear increase in mean ‘% activity impairment’ from 33.6% to 57.8% in carers of patients with prodromal and severe dementia, respectively (Fig 2). For each of the three work-specific WPAI outcomes, highest mean % impairment was reported in the moderate or severe subgroups and caregivers of patients with moderate disease were consistently more impaired than caregivers of mild disease patients.

### Regression analysis

After adjusting for potentially confounding patient and caregiver factors, regression analysis indicated that increasing severity of patients’ CI had a negative impact on measures of caregivers’ health status and degree of burden (Table 2). The number of patients included in the regression analysis was reduced (n = 898) compared with the overall sample due to missing data contained within some of the covariates used.

**Table 2:** Association between severity of dementia and caregiver outcomes—Adjusted analysis.

Covariates	EQ-5D (3L)	EQ-VAS	ZBI	Non-professional hours of care required (per week)	Activity Impairment
**Cognitive impairment stage**					
Prodromal to Mild	-0.018 (-0.064,0.027)	-2.503 (-6.683,1.677)	3.253 (-1.359,7.865)	-2.351 (-17.964,13.263)	2.166 (-4.547,8.880)
Mild to Moderate	-0.033** (-0.057,-0.009)	-3.570** (-5.779,-1.362)	5.159*** (2.722,7.596)	15.406*** (7.156,23.655)	10.718*** (7.171,14.265)
Moderate to Severe	-0.02 (-0.062,0.022)	-5.719** (-9.587,-1.851)	4.325* (0.057,8.593)	28.947*** (14.499,43.394)	7.180* (0.967,13.393)
**Caregiver age, years**	-0.004*** (-0.005,-0.002)	-0.148* (-0.262,-0.033)	0.170** (0.044,0.297)	0.118 (-0.310,0.546)	0.202* (0.018,0.386)
**Male caregiver**	0.025* (0.001,0.050)	1.496 (-0.755,3.746)	-2.741* (-5.224,-0.258)	2.552 (-5.854,10.957)	-2.6 (-6.214,1.015)
**Body mass index, kg/m**^**2**^	0.003* (0.000,0.006)	0.131 (-0.128,0.390)	-0.171 (-0.457,0.114)	-0.054 (-1.021,0.914)	-0.226 (-0.642,0.190)
**Concomitant conditions**					
Myocardial infarction	0.02 (-0.019,0.059)	1.549 (-2.033,5.131)	-1.404 (-5.356,2.548)	-12.017 (-25.396,1.363)	0.238 (-5.515,5.991)
Congestive heart failure	-0.019 (-0.068,0.029)	-1.26 (-5.738,3.217)	3.604 (-1.336,8.544)	10.502 (-6.221,27.226)	6.669 (-0.522,13.860)
Peripheral vascular disease	-0.02 (-0.064,0.025)	2.622 (-1.456,6.701)	0.637 (-3.863,5.137)	14.182 (-1.052,29.416)	0.653 (-5.897,7.204)
Cerebrovascular disease	-0.032 (-0.071,0.007)	-2.186 (-5.765,1.392)	2.58 (-1.368,6.529)	16.793* (3.426,30.160)	7.115* (1.367,12.863)
Hypertension	0.017 (-0.008,0.041)	1.919 (-0.330,4.168)	-3.610** (-6.091,-1.128)	-9.900* (-18.301,-1.500)	-4.442* (-8.054,-0.829)
Transient ischaemic attacks	0.004 (-0.040,0.048)	1.938 (-2.139,6.015)	-2.868 (-7.367,1.631)	10.708 (-4.521,25.937)	-4.354 (-10.903,2.194)
Stroke	-0.027 (-0.077,0.023)	-4.378 (-8.959,0.204)	4.081 (-0.974,9.136)	-6.663 (-23.775,10.450)	5.01 (-2.348,12.369)
**Number of concomitant medications**	-0.012*** (-0.019,-0.005)	-0.754* (-1.400,-0.107)	0.823* (0.110,1.537)	3.017* (0.601,5.432)	0.729 (-0.309,1.768)
**Relationship of caregiver to patient**					
Partner/Spouse (ref.)	-	-	-	-	-
Son/Daughter	0.006 (-0.030,0.042)	0.789 (-2.551,4.128)	1.372 (-2.313,5.057)	-7.863 (-20.337,4.611)	2.046 (-3.318,7.409)
Other	0.025 (-0.016,0.065)	0.353 (-3.384,4.090)	-1.896 (-6.020,2.227)	-10.501 (-24.460,3.457)	-4.671 (-10.674,1.331)
**Caregiver employment status**					
Retired (ref.)	-	-	-	-	-
Working (full/part time)	0.007 (-0.030,0.044)	4.790** (1.387,8.192)	1.444 (-2.310,5.198)	-2.362 (-15.071,10.347)	-4.366 (-9.831,1.099)
Other	-0.032 (-0.067,0.003)	0.236 (-3.006,3.478)	3.462 (-0.115,7.039)	4.532 (-7.577,16.640)	3.032 (-2.174,8.239)
**Country**					
France (ref.)	-	-	-	-	-
Germany	0.126*** (0.090,0.161)	2.335 (-0.952,5.621)	-1.702 (-5.328,1.924)	17.886** (5.610,30.162)	-3.285 (-8.564,1.993)
Italy	0.080*** (0.042,0.117)	0.25 (-3.217,3.717)	-1.39 (-5.215,2.435)	71.123*** (58.174,84.073)	0.072 (-5.497,5.640)
Spain	-0.031 (-0.073,0.011)	-4.581* (-8.484,-0.678)	5.384* (1.077,9.690)	94.469*** (79.889,109.048)	10.278** (4.008,16.547)
UK	0.013 (-0.021,0.047)	2.864 (-0.286,6.015)	-1.293 (-4.769,2.183)	12.564* (0.797,24.332)	5.973* (0.913,11.033)
USA	0.069*** (0.035,0.104)	7.091*** (3.925,10.257)	-2.949 (-6.443,0.544)	38.445*** (26.620,50.271)	-2.507 (-7.592,2.579)

Based only on patients with complete data for all outcomes, exposures and covariates (n = 898). Coefficient between severity groups are additive.

p<0.05.

** p<0.01.

*** p<0.001.

Covariates relate to the patient unless indicated otherwise. EQ-5D (3L), EuroQol 5-dimensions 3-level; EQ-VAS, EuroQol Visual Analogue Scale; ZBI, Zarit Burden Interview; Ref., Reference Case.

For numeric covariates, the coefficient represents the increase (or decrease) in the dependent variable per unit increase (or decrease) in the covariate.

For nominal covariates, the coefficient represents an increase (or decrease) in the dependent variable compared to the reference case for the covariate.

None of the outcomes assessed differed significantly between caregivers of patients with mild dementia versus caregivers of patients with prodromal dementia. However, there were statistically significant differences between caregivers of patients with moderate versus mild dementia across all outcomes assessed: EQ-5D-3L (coefficient: -0.03; p<0.01), EQ-VAS (-3.57; p<0.01), ZBI (5.16; p<0.001), number of non-professional caregiver hours required per week (15.41; p<0.001), and caregiver activity impairment (10.72; p<0.001). All outcomes assessed, with the exception of EQ-5D-3L, differed significantly between caregivers of patients with severe dementia versus caregivers of patients with moderate dementia: EQ-VAS (-5.72; p<0.01), ZBI (4.33; p<0.05), number of non-professional caregiver hours required per week (28.95; p<0.001) and caregiver activity impairment (7.18; p<0.05).

These results indicate that the EQ-5D-3L utility index and EQ-VAS of caregivers of severe dementia patients decrease by 0.07 and 11.79 units, respectively, compared to caregivers of prodromal dementia patients. In addition, the ZBI score increases by 12.74 units, an additional 46.70 hours of non-professional care are required per week, and activity impairment increases by 20.06 percentage points.

With regards to the other covariates included, for each unit increase in caregiver age, the EQ-5D-3L and EQ-VAS decreased by 0.004 (p<0.001) and 0.148 (p<0.05) units, respectively; whereas, burden and activity impairment increased (ZBI: 0.170, p<0.01 and activity impairment: 0.202, p<0.05). An increase in the number of concomitant medications was also associated with worse outcomes, with the exception of activity impairment, and country differences were observed across all outcomes assessed; predominantly in EQ-5D-3L and hours of non-professional care required per week.

## Discussion

On the basis of the raw, unadjusted data from 1,201 caregiver self-assessment forms, measures of caregiver burden (EQ-5D-3L, EQ-VAS, ZBI, number of caregiver hours, and WPAI activity impairment scores) showed statistically significant deterioration (ie, increased caregiver burden) with increasing severity of patients’ dementia, across the full range of disease severities from prodromal to severe disease. Similar trends were seen when data were adjusted for potentially confounding patient and caregiver characteristics; for example, caregiver age, number of concomitant medications the patients was receiving, and country.

Dementia incidence and CI deteriorates with age [2], so it is not unexpected that the mean age of caregivers in the current study (63.9 years) was approaching retirement age. Indeed, more than half of caregivers were retired (49.5%) or unemployed (6.2%). In the GERAS study [10], an even lower proportion (23.4%) of patients were working for pay, although this was in a slightly older (mean 67.3 years) population than the current study. Most (69.1%) caregivers in the current study were female and most were close family members of the patient; 24.6% were daughters, although only 8.0% of caregivers were patients’ sons. Where the caregiver was the patient’s spouse, there were more female caregivers with an approximately 60/40 split for most dementia severities and 65.3% female spousal carers in the severe subgroup. This is consistent with the literature, where the typical profile of a dementia caregiver is that of a middle-aged or older female child or spouse of the patient [10,16], indicating that the caregiver sample recruited within the DSP are a representative population of caregivers.

Unadjusted mean EQ-5D-3L questionnaire scores decreased from 0.869 for caregivers of patients with prodromal CI to 0.807 for caregivers of patients with severe dementia. In an assessment of the use of the EQ-5D-3L across multiple studies, Walters and Brazier, 2005 [31] reported a mean minimally important difference for the EQ-5D questionnaire to be 0.074. On the basis of this criterion, the current difference in EQ-5D-3L between carers of patients with prodromal versus severe dementia represented a clinically meaningful deterioration in health status with increasing disease severity. Regression analysis of EQ-5D-3L scores showed a statistically significant deterioration in health state for caregivers of patients with mild compared with moderate dementia. No statistically significant differences between prodromal versus mild and moderate versus severe dementia were evident but a numerical trend remained for deterioration in EQ-5D-3L score with increasing disease severity. Regression analysis of EQ-VAS scores did however demonstrate statistically significant decreases in self-perceived health status for caregivers of patients with mild versus moderate and moderate versus severe disease.

Analysis of unadjusted ZBI scores indicated a “mild to moderate burden” in caregivers of patients with prodromal to moderate dementia, increasing to a “moderate to severe” burden in caregivers of severe dementia patients [26]. Regression analysis showed a statistically significant difference in ZBI scores between carers of mild versus moderate and moderate versus severe disease, with a numerical trend towards increased burden between prodromal and mild disease severity subgroups. The observed association between increasing ZBI score and increasing disease severity reflects the higher perceived burden and lower quality-of-life experienced by caregivers as patients’ CI progresses and is consistent with findings of increased caregiver burden (measured by the ZBI) with increased MMSE (p<0.001) in the GERAS study [20].

The number of caregiver hours was also shown to increase with patients’ disease severity. Based on unadjusted data, there was a statistically significant increase in the mean number of caregiver hours required per week, ranging up to 108.6 hours for carers of patients with severe dementia. The regression analysis of unadjusted data showed that the required amount of non-professional caregiver time increased by a total of 46.7 hours when caring for patients with severe compared with prodromal dementia, with statistically significant increases in weekly required hours seen when carers of patients with mild versus moderate and moderate versus severe dementia were compared. Of note, even for patients with prodromal disease, the mean number of hours required for caregiving was quite substantial (36.9 hours per week) which highlights the weight of the issue of caring for someone in this position. The number of hours of caregiving time required represents perhaps the most tangible measure of caregiver burden. If raw data are averaged over 7 days, caregiving time ranges from ~5 hours to in excess of 15 hours per day dependent on disease severity. In the GERAS study [10], monthly caregiver hours also increased significantly in France, Germany, and the UK, ranging from 83.4 to 147.5 hours per month for caregivers of patients with mild disease, to 258.0 to 320.8 hours per month for caregivers of patients with moderately severe/severe disease. It is clear that, regardless of other aspects of health status, caregiving can be similar to a full-time job. Even in the less than one quarter of caregivers who were able to maintain paid work in the GERAS study, usual working hours were reported to decrease by approximately 12 hours per week and an average of 1 day of work per month was lost.

Unadjusted WPAI data indicated that carers of patients with moderate or severe dementia experienced the greatest degree of activity impairment. Mean ‘% activity impairment’ increased directly in relation to patients’ disease severity across all subgroups: impairment of 9.8%, 28.6%, 35.9% and 57.8% in carers of prodromal, mild, moderate, and severe patients, respectively. In the adjusted regression analysis mean ‘% activity impairment’ was 20.1 percentage points greater when caring for severe versus prodromal dementia, with statistically significant deterioration seen when comparing carers of mild versus moderate (p<0.001) and moderate versus severe (p<0.05) patients. Other aspects of the WPAI could not be subjected to regression analysis because of too few caregivers in paid employment and thus not completing the questionnaire. For the unadjusted analysis of the other three WPAI measures, ‘% overall work impairment’, ‘% presenteeism’, and ‘% absenteeism’ also increased directly in relation to patients’ disease severity across all subgroups; the one exception being for ‘% absenteeism’, which indicated that caregivers of patients with moderate disease were more impaired than caregivers of severe disease (37.4% and 33.6%, respectively). The overall measure of ‘mean % activity impairment’ (which is the only non-work-specific measure) is the only measure for which a sufficiently large data sample was collected to eliminate spurious trends.

Despite the clear trend towards increased caregiver burden with increasing severity of patients disease, it is notable that, there were no statistically significant differences for carers of patients with mild versus prodromal disease with any measure, or for severe versus moderate disease with the EQ-5D-. It would be interesting to investigate why many of the adjusted analyses were non-significant in further research; particularly focusing on confounding factors which may have contributed to the loss of significance.

There are a number of technical limitations of the DSP methodology. Most importantly, as the study was based on cross-sectional rather than longitudinal survey data and included all dementia subtypes, the data may be used to assess the association between factors but not to assess causality. The sample collected was pseudo-random rather than truly random; as data were collected from consecutively consulting patients, the sample was more likely to include patients that consult more frequently and therefore possibly have more severe disease. On the other hand, patients at the more severe/advanced stages of the disease may not currently be in standard care pathways or may have professional rather than non-professional caregivers, and thus would have been excluded from this survey. Also, the methodology relied on the accurate reporting of data by physicians and caregivers and there may have been an element of recall bias for information about past events. Missing data were expected, for example due to imperfect or incomplete physician knowledge of patients’ medical history and unwillingness of some caregivers to answer specific questions. Furthermore, the number of patients included in the regression analysis was reduced compared with the overall sample as some of the covariates used contained missing data. Additionally, as completion of caregiver forms was voluntary, not all patients had a corresponding caregiver form. To assess whether this approach introduced any bias into this analysis, basic demographics and clinical characteristics were compared between patients with and without a corresponding caregiver form (Supplementary S1 Table). The proportion of female patients and white/Caucasian patients were consistent between the two subgroups of patients. However, patients for whom a caregiver form was supplied were on average older, more likely to be retired, had been diagnosed with dementia for a longer period of time and had a worse MMSE score compared to patients without a corresponding caregiver form (all p<0.001). Although the observed differences were statistically significant, this may have been driven by the large sample size as the magnitude of the differences were relatively small. The smaller proportion of forms collected from caregivers of patients with less severe disease may, to some degree, reflect lower requirement for caregiver support at this stage. However, sampling bias cannot be eliminated and the number of forms collected in a particular subgroup should not be considered as a robust indicator of actual caregiver requirement. Likewise, the consistent significant findings between the mild and moderate dementia groups might be owing to the larger samples sizes obtained for these subgroups, which again could have affected the results.

For all patients, their most recent MMSE score was used to categorise their disease severity, which included a prodromal category (score of 27–30). There has been some debate about how to distinguish the prodromal stages of a pathological disease compared with ‘normal’ age-related changes in cognition, which suggests that the definition used in this study may not be sufficient. However, in real-world clinical practice there is a lack of standard testing instruments for CI and thus the MMSE is possibly the most commonly used short assessment tool for providing an overall measure and therefore the best that is currently available. Finally, this study focused on the association between severity of CI and caregiver burden; however, it should be noted that other characteristics, such as the behavioural disturbance/ neuropsychiatric symptoms of the patient, have also found to be strong determinants of caregiver burden [19]. Furthermore, the survey did not collect data on educational level, a relevant omission as it is known to be associated with cognitive function [32,33,34].

Whilst acknowledging these limitations, the data collected do provide a substantial body of data (N = 1,201) in a representative, real-world population of caregivers of patients across the full range of disease severities, from prodromal to severe dementia. Furthermore, the Dementia DSP has collected data on a number of different outcomes related to caregiver burden using different validated instruments and the overall pattern of increasing caregiver burden with increased severity of patients’ CI is clear. Individual, validated instruments are often created to assess utility across a range of diseases rather than one specific disease, and therefore may be found to be more or less sensitive when applied to a specific situation such as caregiving for dementia. However, the findings of increased burden with increased disease severity in the current study were seen across a number of outcome measures and using different instruments, thus strengthening the body of data and lending confidence to the overall conclusion. These data therefore provide a valuable addition to the current literature and understanding of the relationship between caregiver burden and the severity of patients’ disease. This study goes beyond the GERAS and ICTUS caregiver analyses as it represents an up-to-date focused analysis in a large number of patients with a broader range of CI (ie, prodromal to severe impairment compared with mild to severe AD in GERAS and mild to moderate AD in ICTUS) and in a larger number of countries (ie, five European countries and the US compared with GERAS and ICTUS both of which only included limited European countries).

## Conclusions

On the basis of a large body of real-world data collected from 1,201 non-professional caregivers of patients with dementia, this study confirmed an association between increased caregiver burden and increased severity of patients’ disease by analysis of a wide range of validated measures of caregiver burden, including health state, ability to perform work and home activities, number of caregiver hours, and caregivers’ self-perception of their health state. It is important to study the factors that affect caregiver burden so that the appropriate professional help and assistance can be provided to improve both caregiver health and wellbeing and also the quality of care given to dementia patients. These findings suggest that maintaining patients in the earliest (prodromal or mild) stages of dementia for as long as possible may potentially play a role in protecting caregiver wellbeing, although further research is required to confirm and strengthen this hypothesis.

## Supporting information

S1 Table Patient demographics. (DOCX)
