# Supplementary material for: Non-professional caregiver burden is associated with the severity of patients’ cognitive impairment
Source: PLoS One. 2018 Dec 6;13(12):e0204110. doi: 10.1371/journal.pone.0204110 (PMC6283568; doi:10.1371/journal.pone.0204110)
Supplement: S1 Table — (DOCX) [file pone.0204110.s001.docx]

**Supplementary Tables**

**S1 Table. Patient demographics**

|  | **Overall (n=5,166)** | **Caregiver form not available (n=3,965)** | **Caregiver form available (n=1,201)** |
| --- | --- | --- | --- |
| **Patient age^a^ , years ***** |  |  |  |
| Mean (SD) | 75.7 (8.7) | 75.2 (8.9) | 77.4 (7.9) |
| Median (IQR) | 77.0 (70.0-82.0) | 76.0 (69.0-82.0) | 78.0 (72.0-83.0) |
| **Female^b^** | 2,745 (53.2) | 2,123 (53.6) | 622 (51.8) |
| **White/Caucasian^c^** | 4,459 (86.8) | 3,413 (86.4) | 1,046 (87.9) |
| **Employment status^d ***^** |  |  |  |
| Working full time | 139 (2.7) | 126 (3.2) | 13 (1.1) |
| Working part time | 131 (2.6) | 124 (3.2) | 7 (0.6) |
| Homemaker | 491 (9.6) | 397 (10.1) | 94 (7.9) |
| Student | 6 (0.1) | 6 (0.2) | 0 (0) |
| Retired | 4,120 (80.6) | 3,110 (79.2) | 1,010 (85.0) |
| Unemployed | 226 (4.4) | 162 (4.1) | 64 (5.4) |
| **Duration of disease^e^ , months ***** |  |  |  |
| Mean (SD) | 28.2 (31.9) | 26.4 (30.7) | 34.2 (34.9) |
| Median (IQR) | 18.0 (7.0-39.0) | 16.0 (6.0-37.0) | 25.0 (9.0-49.0) |
| **Most recent MMSE score ***** |  |  |  |
| Mean (SD) | 20.3 (5.8) | 20.7 (5.7) | 18.9 (6.0) |
| Median (IQR) | 21.0 (17.0-25.0) | 22.0 (18.0-25.0) | 20.0 (16.0-23.0) |

Data are n (%) unless otherwise indicated. SD, Standard Deviation; IQR, Interquartile Range

*** p<0.001

^a^ Overall, 3 missing responses; Form not available, 2; Form available, 1.

^b^ Overall, 8 missing responses; Form not available, 7; Form available, 1.

^c^ Overall, 26 missing responses; Form not available, 15; Form available, 11.

^d^ Overall, 53 missing responses; Form not available, 40; Form available, 13.

^e^ Overall, 123 missing responses; Form not available, 95; Form available, 28.
